# Effects of Social Network Exposure on Nutritional Learning: Development of an Online Educational Platform

**DOI:** 10.2196/games.4002

**Published:** 2015-10-05

**Authors:** Noa Dagan, Daniel Beskin, Mayer Brezis, Ben Y Reis

**Affiliations:** ^1^ Braun School of Public Health Hebrew University School of Medicine Jerusalem Israel; ^2^ Predictive Medicine Group Computational Health Informatics Program Boston Children's Hospital Boston, MA United States; ^3^ Predictive Medicine Group Computational Health Informatics Program Boston Children's Hospital and Harvard Medical School Boston, MA United States

**Keywords:** nutrition requirements, obesity, public health, social networking sites

## Abstract

**Background:**

Social networking sites (SNSs) such as Facebook have the potential to enhance online public health interventions, in part, as they provide social exposure and reinforcement.

**Objective:**

The objective of the study was to evaluate whether social exposure provided by SNSs enhances the effects of online public health interventions.

**Methods:**

As a sample intervention, we developed Food Hero, an online platform for nutritional education in which players feed a virtual character according to their own nutritional needs and complete a set of virtual sport challenges. The platform was developed in 2 versions: a "private version" in which a user can see only his or her own score, and a "social version" in which a user can see other players’ scores, including preexisting Facebook friends. We assessed changes in participants’ nutritional knowledge using 4 quiz scores and 3 menu-assembly scores. Monitoring feeding and exercising attempts assessed engagement with the platform.

**Results:**

The 2 versions of the platform were randomly assigned between a study group (30 members receiving the social version) and a control group (33 members, private version). The study group's performance on the quizzes gradually increased over time, relative to that of the control group, becoming significantly higher by the fourth quiz (*P*=.02). Furthermore, the study group's menu-assembly scores improved over time compared to the first score, whereas the control group's performance deteriorated. Study group members spent an average of 3:40 minutes assembling each menu compared to 2:50 minutes in the control group, and performed an average of 1.58 daily sport challenges, compared to 1.21 in the control group (*P*=.03).

**Conclusions:**

This work focused on isolating the SNSs' social effects in order to help guide future online interventions. Our results indicate that the social exposure provided by SNSs is associated with increased engagement and learning in an online nutritional educational platform.

## Introduction

### Online Public Health Interventions

With the significant amount of time people spent engaging with digital media [1], the Internet presents an ideal opportunity for health education. Research on Internet-delivered public health interventions is an emerging field that has gained momentum in recent years [2]. While most studies of online interventions (computer games, Internet sites, Facebook applications; mobile apps, etc) have focused on evaluating the overall effect of the intervention, very few studies have tried to isolate the effects of specific intervention characteristics [2,3].

### Leveraging Social Networking Sites for Public Health Purposes

Social networking sites (SNSs) are a major component of Internet use by young adults [4], partly due to their ability to engage the human need for social reinforcement [5]. The use of these networks involves an element of “social exposure,” in which users observe and exchange feedback on one another's activities. For example, social exposure has been used to successfully and dramatically increase organ donation registration [6]. The specific impacts of social connections on weight [7], and of social support on obesity preventing behavior [8] have been previously demonstrated outside the framework of SNSs.

Currently, the most popular SNS in the world is Facebook [9], which reports over 1.2 billion active users [3]. As much as 57% of American adults have a Facebook account, with each individual connected to an average of 338 friends in the network [10].

There is a tremendous opportunity to leverage the potential of SNSs to promote public health issues in general, and obesity prevention in particular. Obesity is associated with many of the most common and costly medical problems in Western society [11,12], reaching epidemic proportions and affecting roughly one-third of US young adults aged 20-39 [13]. In light of these alarming trends, there is a critical need for interventions aimed at preventing obesity in young adults. Although the direct association between nutritional knowledge and dietary behavior is debated [14], it is plausible that such knowledge is required once an individual aspires to improve his or her nutrition. A statement by the American Heart Association argues that social networks may be critical to shaping young people’s eating behaviors, and emphasized the scarcity of interventions targeting SNSs [15].

A literature review from 2010 identified only one controlled intervention study on social media and health outcomes [16]. There are 2 systematic reviews published recently that found only 16 studies overall exploring the influences of SNSs on health behavior change [17,18]. Most of these studies reported some significant influence, but with considerable heterogeneity. Yet, the vast majority of these studies evaluated the overall effectiveness of an intervention involving a component of SNSs, but did not isolate the specific effect of social exposure within the SNSs.

### Objective

The goal of this study is to evaluate whether the social exposure provided by SNSs can increase the effect of online public health interventions, specifically by evaluating its influence on the learning curve for nutritional knowledge.

## Methods

### The Food Hero Platform for Nutrition Education

In order to conduct this study, we developed a game-based educational platform called *Food Hero*, focused on nutrition education. The Facebook network was chosen as the SNS for developing the app due to its widespread popularity [3].

**Figure 1:**
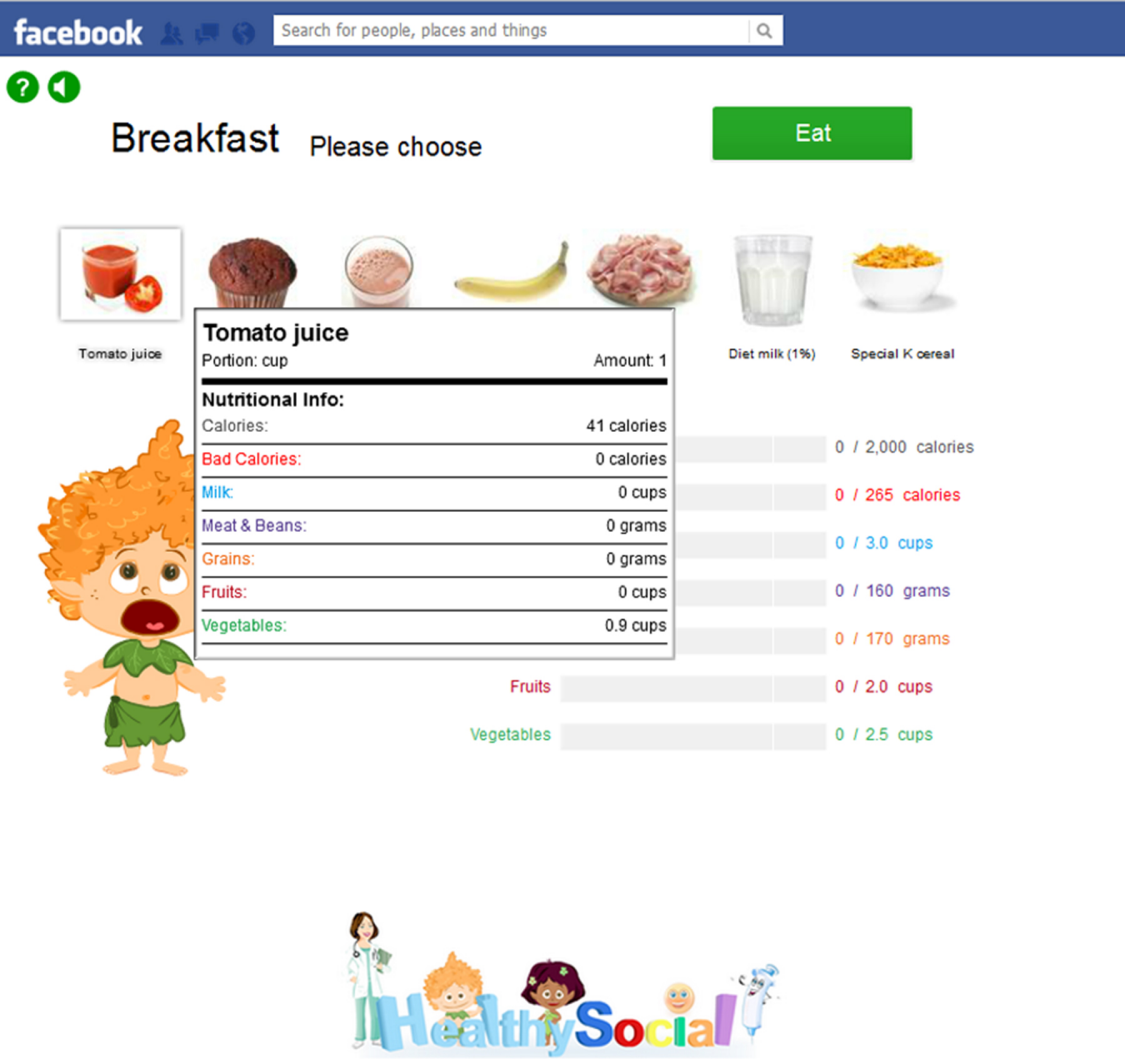
Feeding screen of Food Hero guided by nutritional information cards.

**Figure 2:**
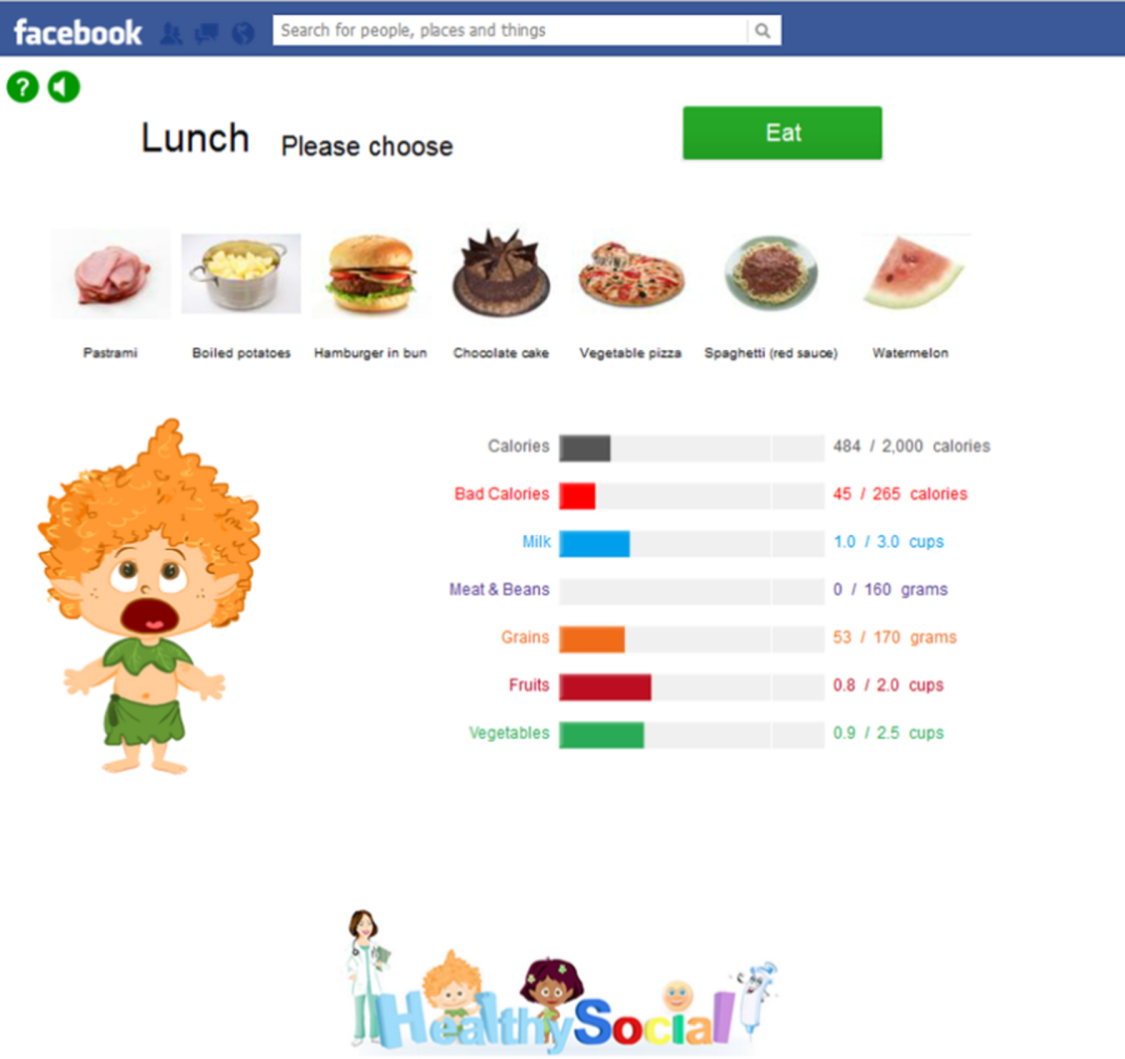
Feeding screen of Food Hero guided by status bars.

In the *Food Hero* platform, the user begins by choosing a virtual character. During each game day, the user must assemble an optimal food menu for the virtual character, based on the user’s real-world caloric and nutrient composition needs (calculated according to the user's sex, age, weight, and exercise habits). The user is presented with a selection of food items for each of 3 meals and 1 snack, along with detailed nutritional information cards for each item (Figure 1). During menu selection, the user is able to monitor the progress of daily requirements through a set of status bars (Figure 2). After assembling the menu, the user receives feedback on his or her performance, including a numerical score of performance-based points. The user may perform repeated attempts to select the optimal menu for each game day, until the user is satisfied. At the end of each game day, the user is given the opportunity to complete a set of virtual running, cycling, and swimming challenges. The character's ability to complete these challenges is directly dependent on both the speed with which the user presses specific keys on the keyboard in order to make the character move and the quality of that day's menu selections; too much or too little food, or an imbalanced diet will slow the character down in the sports challenges. The user can also use accumulated performance points to acquire accessories for the virtual character, thus further assisting in completing the various sports tasks.

### Study Groups

**Figure 3:**
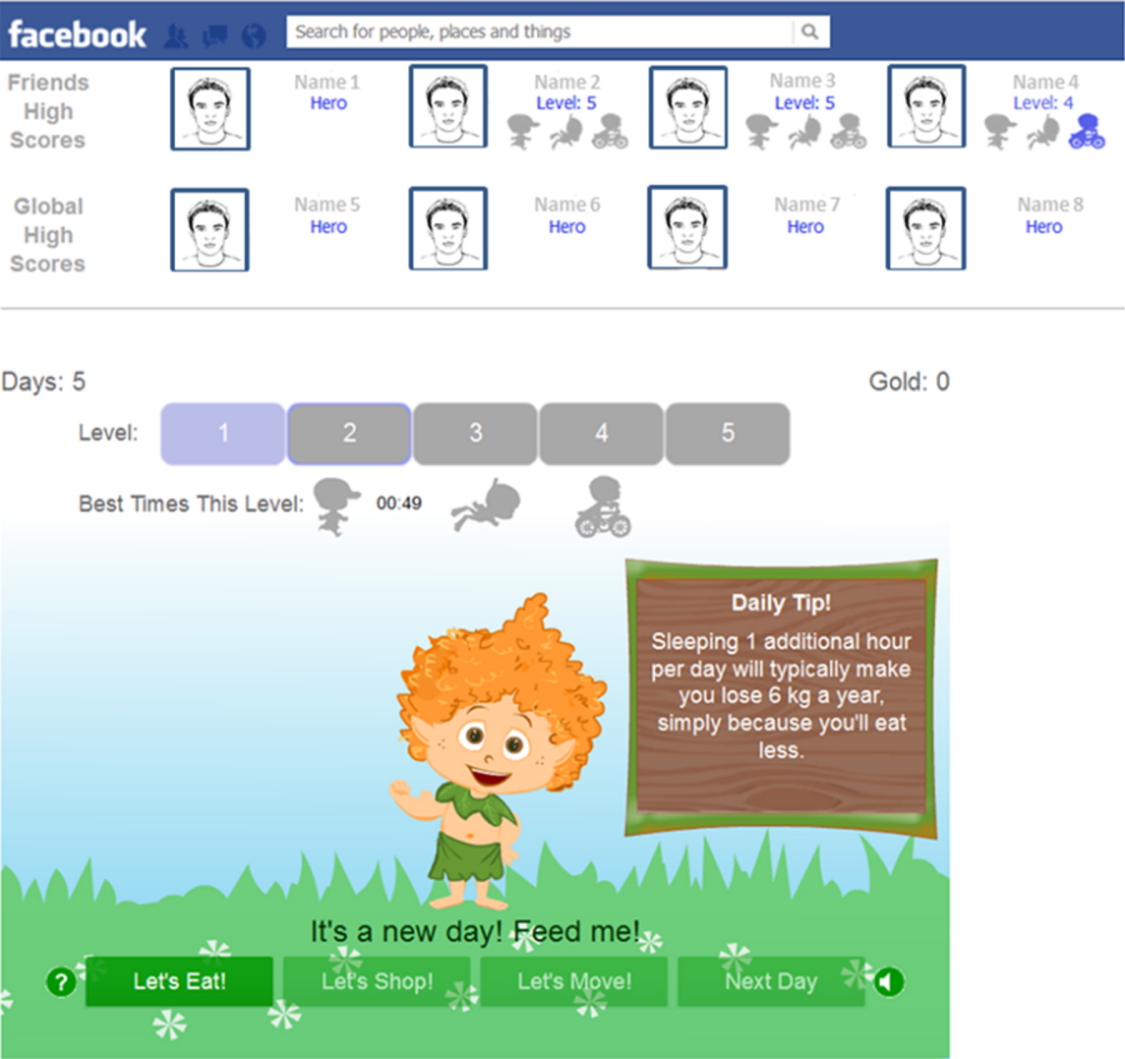
Home screen of the social version, showing the high-score bars.

We developed 2 different versions of the platform, a “private version” and a “social version,” and assigned these versions randomly to different users. In the private version, only the user's own score is presented, without any information about the performance of other players. In the social version, the user’s performance is presented in the context of other players, a high-score bar shows the scores of the 5 best players, while another shows the top 5 scores from within the user's Facebook friends (Figure 3). The social version also shows pop-up messages any time one of the user's friends successfully completes a level. Users randomly assigned to the social version comprised the study group, whereas users randomly assigned to the private version formed the control group. Additional screen shots of the *Food Hero* platform are provided in Multimedia Appendices 1-13.

### Nutritional Information

Nutritional information was obtained from the US Department of Agriculture's official database for dietary guidelines, “My Pyramid.” This information included the definition of the 5 food groups, a list of food items along with their nutritional value, and the formulas to calculate the proper intake of calories and desired level for each food group (adjusted for sex, age, weight, and exercise habits) [19,20].

### Study Participants and Recruitment Process

Participants were Facebook users who chose to install the platform and agreed to join the study by completing a consent form presented as part of the app installation process. For reasons of legal consent, all study participants were older than 18 years of age, as stated in the participation consent form. Exclusion criteria included users that did not provide consent to participate in the study, users that did not report their age or reported an age younger than 18 years, and users that installed the software, but did not actively start to use it (ie, did not complete the first game day).

The distribution of the platform took place over a 2-month period, starting in April 2012, and was spread through the SNS by peer-to-peer message dissemination. The distribution was initiated by a single message recommending *Food Hero*, published by a seed individual, one of the research team members, to a group of Facebook friends. The rest of the distribution was based on users recommending the platform to their friends, and on automatic messages published by the platform on users' Facebook walls. This method of distribution was chosen to ensure that study members would have Facebook friends among the study population, expecting that watching the performance of known acquaintances will have more social impact than that of strangers' [21].

### Data Collection

All data for the study were collected electronically within the *Food Hero* platform. During the platform installation process, each user was presented with a consent form for participation in the study, and a personal information form including age, sex, weight (in kilograms/pounds), height (in centimeters/inches), mother tongue, education level, hours of weekly physical activity, and smoking status. To address the research question, it was necessary to track the users' nutritional knowledge throughout the course of the study's follow-up period. For this purpose, we developed 4 quizzes, each containing 8 different multiple-choice questions that had not been seen by the user before, and were based on information introduced within the platform before the relevant quiz. The quizzes were presented to users during game days 2, 6, 10, and 14. A secondary variable to assess user nutritional knowledge was the score of the first menu assembly attempt on 3 fixed game days—days 4, 8, and 12—during which the user was required to build a menu in an unguided manner, without the help of the usual nutritional information cards and status bars. In order to measure user engagement with the educational platform, we recorded the time spent choosing each menu, the number of repeat attempts to build the menu in each game day, and the number of sport challenges the user tried to complete in each game day.

On the 15th game day, at the end of the follow-up period, a final questionnaire was presented to evaluate each user’s impressions of how the SNS influenced his or her use of the platform and the effect the platform had on approach and behavior regarding nutrition. Participants who did not complete the full follow-up period received a request to answer the questionnaire by email. Questions regarding the social network influence were presented to the control group members hypothetically—what effect they would expect if they could have seen their friends' performance. The final questionnaire is provided in Multimedia Appendix 14.

### Statistical Analysis

Descriptive statistics and comparison of groups were performed using SPSS Statistics 18.0. Comparison of quantitative variables between study groups was performed using the *t* test, or the nonparametric Mann-Whitney test (M-W) when the sample size was small and was not normally distributed. The connection between 2 qualitative variables was evaluated using the chi-square test, or the Fisher exact test in cases of limited number of observations in a cell. All the statistic tests were two-tailed, and a *P* value of 5% or less was considered statistically significant.

### Study Approval

The Ethics Committee for Human Studies of the Hebrew University of Jerusalem approved the project.

## Results

### Study Participants

Of the 70 Facebook users who installed the platform successfully during the 2-month distribution period, 7 were excluded from the data analysis (2 did not enter their age, 5 did not start active use of the app). A total of 63 users, of which 30 belonged to the study group and 33 to the control group, were included in the analysis. No significant differences were found between the basic characteristics (age, sex, body mass index; BMI, etc) of the study participants in both groups (Table 1).

**Table 1:** Study participants’ character by study group.

Participants’ character		Study group	Control group
Number		30	33
Age (average in years)		29.0	31.4
Sex (male percentage), n (%)		13/28 (46)	10/33 (30)
BMI^a^ (average)		22.0	23.2
**Mother tongue (rate from study group), n (%)**			
	Hebrew	26/30 (87)	27/33 (82)
	English	2/30 (7)	4/33 (12)
	Other	2/30 (7)	2/33 (6)
**Education level (rate from study group), n (%)**			
	Did not complete/completed high school	3/30 (10)	1/33 (3)
	Studying for/completed first degree	15/30 (50)	14/33 (42)
	Completed graduate degree	12/30 (40)	18/33 (55)
**Physical activity (rate from study group in weekly hours), n (%)**			
	0-0.5	8/30 (27)	7/32 (22)
	0.5-2	6/30 (20)	7/32 (22)
	Over 2	16/30 (53)	18/32 (56)
**Smoking status (rate from study group), n (%)**			
	Nonsmokers	25/30 (83)	28/32 (88)
	Former smokers	3/30 (10)	2/32 (6)
	Current smokers	2/30 (7)	2/32 (6)
Facebook friends playing *Food Hero* (average)		2.9	2.6

^a^BMI was calculated according to height and weight reported by users.

### Application Use

Study participants played an average of 8.8 game days, with no significant difference between persistence rates in both study groups (*P*=.25, *t* test). The full follow-up period of 15 game days was completed by 32% (20/63) of the participants. A total of 40% (25/63) of study participants answered the final questionnaire. Naturally, more quiz grades and unguided menu assembly scores were accumulated for participants that completed more game days. Statistical analysis of these variables included all the participants that reached the game day in which they were examined.

### Change in Nutrition Knowledge by Study Group

**Figure 4:**
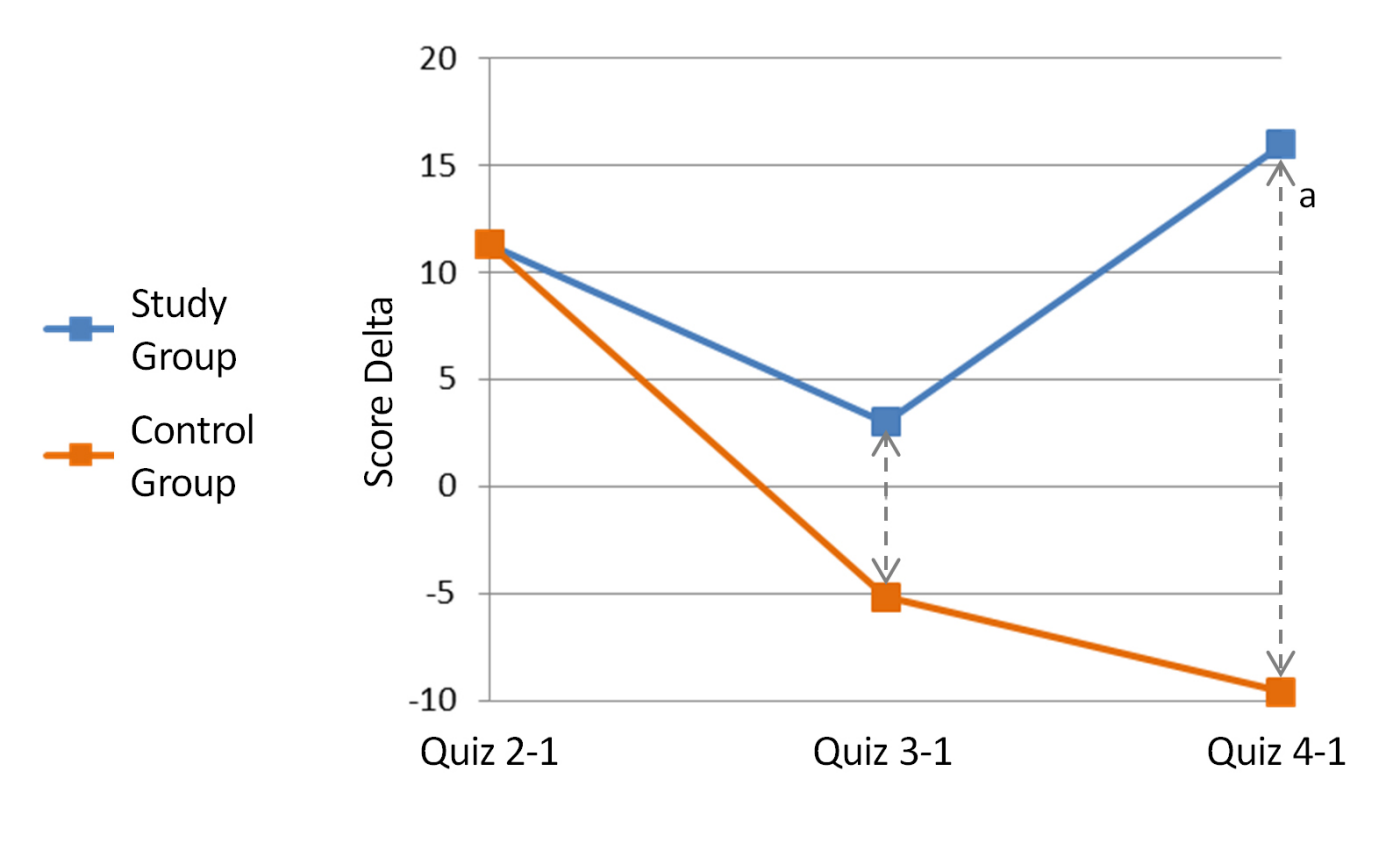
Average quiz grade improvement by study group (relative to the first quiz grade). Each point includes all participants that answered the relevant quiz, in comparison to the grade the same participants received in the first quiz. (a) *P*=.02 (t test).

**Figure 5:**
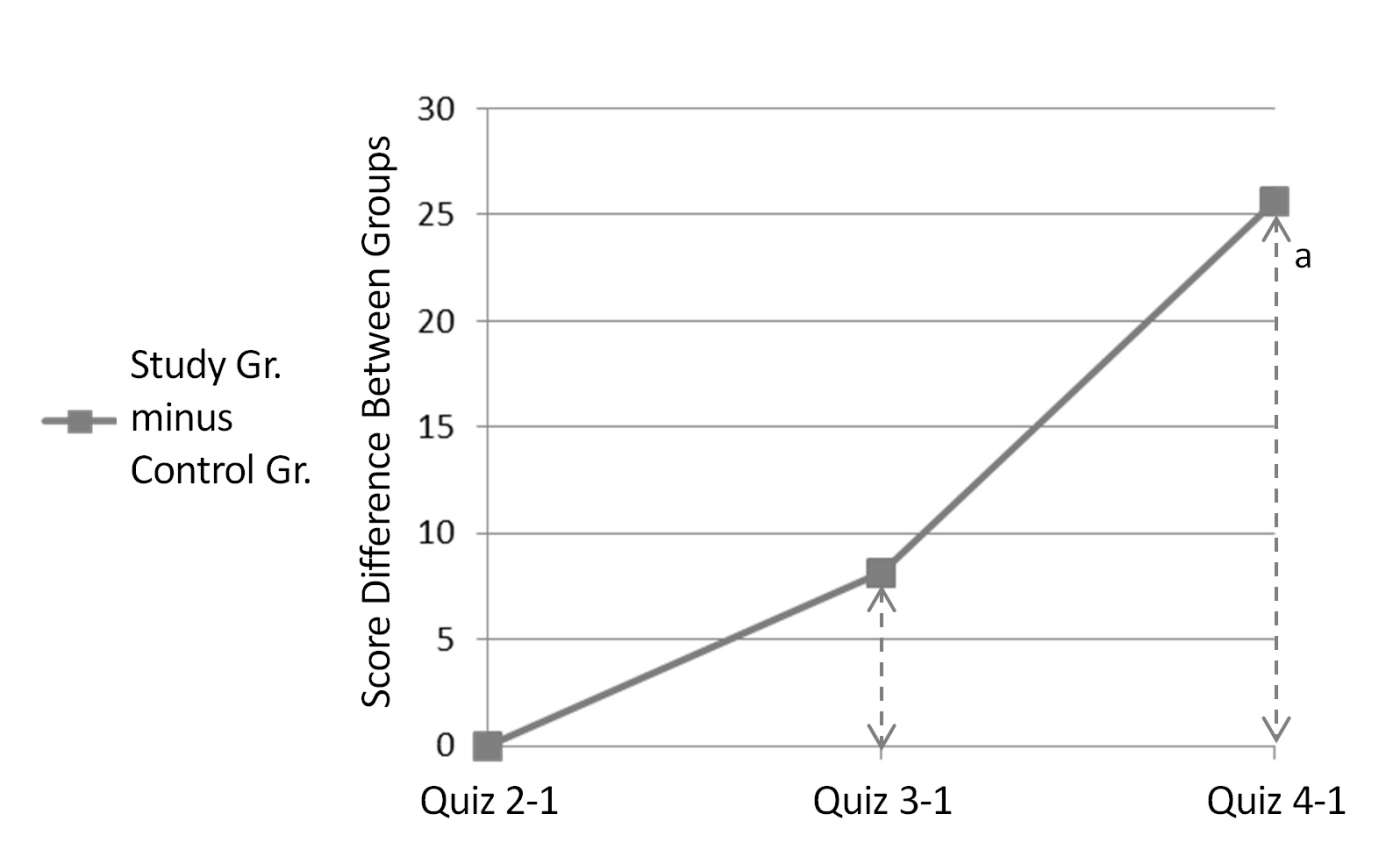
Difference between average quiz grade improvement between study groups. (a) *P*=.02 (t test). Gr.= Group.

As stated, quiz grades were chosen to be the primary variable to assess knowledge change with platform use. The average grade of the first quiz, presented to players in the second game day in order to document the basic knowledge of the users, was practically identical between study groups, with both groups answering 57% (average of 4.6/8 correct answers) of the questions correctly. The later quiz grades were analyzed by calculating a set of quiz grade improvement variables, measuring the improvement of each quiz score relative to the first quiz the player had answered. Analysis of these variables revealed a trend of greater improvement over time among the study group. For control group members, the average of the second quiz improvement variable was positive, meaning improvement relative to the first quiz, but in further quizzes there was a gradual decrease in performance (Figure 4). For members of the study group, however, average scores for quizzes 2-4 were improved relative to quiz 1, with the greatest improvement present in the final quiz (Figure 4). The difference between the improvement variables of both study groups increasingly diverged over time (Figure 5), becoming statistically significant by the fourth quiz (*P*=.02, *t* test).

As with quiz grades, unguided menu assembly scores were analyzed by calculating the improvement relative to the performance of each player on the first unguided menu assembly day. These scores were then converted to standardized z-scores, because the original scores ranged in unlimited scale, including negative numbers. The study group exhibited a positive improvement in both the second and third unguided menu assembly scores, whereas the control group exhibited deterioration in performance over time. The average z-score of the second and third unguided menu assembly days was 0.18 above the first menu assembly day in the study group, compared with -0.26 in the control group.

### Platform Engagement by Study Group

Members of the study group also invested greater time and effort trying to progress through the stages of the educational platform, and they spent an average of 3 minutes and 40 seconds on each menu assembly, as opposed to 2 minutes and 50 seconds in the control group. In addition, study group members performed an average of 1.42 attempts to build the menu on each game day, compared with 1.37 attempts in the control group. The average number of sport challenges the user tried to complete in each game day (reflecting the user's motivation to advance through the game levels) was 1.58 in the study group and 1.21 in the control group (*P*=.03, M-W).

### Participants' Perception of the Social Networking Sites' Effect

The final questionnaire demonstrated that most participants, from both study groups (with no statistically significant difference), perceived that being able to watch other players' performance can encourage engagement with the platform. Overall, 64% (14/22) of respondents expressed a medium or high level of agreement with a statement that they were interested in other players’ performance. A total of 67% (14/21) expressed a medium or high level of agreement that other players’ performance encouraged their engagement with the platform and increased their motivation to succeed. Almost all respondents (95%, 20/21) expressed a low level of agreement with a statement that other players' performance discouraged engagement with the platform.

### Platform Effects on Nutritional Approach and Behavior

The questionnaire also included statements designed to obtain an initial indication of whether *Food Hero* also has effects beyond changes in knowledge. Players' answers suggested that the platform may have the potential to influence individuals’ nutritional approach and behavior (with no statistically significant difference between study groups): 43% (9/21) of all respondents answered that the platform had highly affected their desire to improve their eating habits, and another 38% (8/21) answered they were moderately affected. On questioning whether the platform actually improved eating habits, 32% (7/22) and 45% (10/22) answered they were affected to a high or moderate degree, respectively. Questions exploring specific behavioral changes received the highest levels of positive responses: 73% (16/22) and 55% (12/22) of respondents stated that their attention to food composition and caloric values were highly improved, respectively.

## Discussion

### Principal Results

The results of this study indicate that users of an online educational platform who were exposed to the performance of their friends on the social network exhibited increased improvement in their nutritional knowledge, as well as increased engagement with the platform, compared to those who were not exposed to their friends' performance. It is plausible that these players' greater engagement with the platform is due not only to their ability to see their peers' performance, but also to their understanding that their performance is equally visible to their peers.

Many studies have examined the potential correlation between nutritional knowledge and dietary behavior, with many studies reporting that no such correlation was found [14]. Although not the main purpose of this study, we attempted to obtain an initial indication of whether an educational platform like *Food Hero* could also potentially lead players to improve their nutritional habits. A substantial rate of respondents reported that the platform positively affected their desire to improve their eating habits, and positively affected their actual eating habits. Although it is widely accepted that the reliability and validity of self-reported health habits is limited [22,23], these results encourage further research on the effects of this educational platform and SNSs in general on changes in eating habits.

### Comparison With Prior Work

Although using online social media for promoting public health has been increasingly studied in recent years [17,18,24], we found very few studies that tried to characterize which specific factors make online public health interventions successful. Specifically, we found that most SNS health-related studies did not isolate the social effect of the SNS. Bramlett et al [25] found that a Facebook page had greater impact on food-safety attitudes and practices, compared to a traditional lecture, but did not study the SNS's effects as opposed to other online interventions. Graham et al [26] did compare 2 online interventions for smoking cessation, but the arm of the study that included a social network also included other added elements such as tailored content, thus masking the isolated effect of the SNS. Cavallo et al [27] attempted to isolate the effect of the SNS. They compared the effect of an educational website encouraging physical activity to a combination of the website with a Facebook group meant to provide support. This study did not find an added effect of the Facebook group, a fact the writers partially attribute to the participants' recruitment process that did not include individuals along with a subset of their existing friends. In our study, participant recruitment occurred using peer-to-peer messaging, and thus ensured that each participant had an average of 2.75 Facebook friends enlisted in the study, which may have enhanced the social element and contributed to the difference between the study groups. Foster et al [28] did manage to isolate the SNS's effect and demonstrate its advantage by comparing 2 groups of 5 formerly acquainted nurses using a pedometer, with and without the ability to see the number of steps performed by their peers. We expect that characterization of specific successful elements of online interventions, as we attempted to do, will be the focus of more future studies. A study is currently being conducted by Cobb et al [29] to study the factors affecting the diffusion of an online intervention for smoking cessation through Facebook.

### Limitations

There are several limitations of this study. First, we did not focus on broad participant recruitment, but rather on the natural diffusion of the app through Facebook. The effects were large enough to produce statistically significant differences between study groups, and future work will further study factors that increase the distribution of the platform, building on relevant prior work such as that of Cobb et al [29]. The authors recognize that developing a successfully “viral” online product is a challenge even for commercial organizations such as professional game companies, so realistic expectations for a scientific research project are set accordingly. Second, our study population was relatively homogeneous in terms of education level and native language, probably because the app was distributed by peer-to-peer messaging to ensure that participants had existing friends in the study. Finally, due to requirements of research consent, the study excluded participants under the age of 18. Future studies will explore ways to study younger populations such as teenagers within the consented research framework.

### Conclusions

In this study, we sought to evaluate whether SNS exposure can be used to enhance online public health interventions by isolating the effects of the SNS component. The results indicate that when people have the ability to see the performance of their peers, and assume their performance is similarly exposed to their peers, the engagement with the online nutritional educational platform increases, and they gain more knowledge in the process. These findings strengthen the motivation to leverage the enormous time spent on SNSs for beneficial purposes such as health promotion. Further research is needed to include more participants from heterogeneous populations and other age groups in order to increase external validity, and to assess the effect of SNSs on actual behavioral change. While not every attempt at online health promotion intervention will gain popularity and become widely used, once the initial investment is made and a successful intervention is developed, the potential number of individuals impacted can be very large. Therefore, understanding how to maximally leverage the power of social networks to make online interventions as effective as possible has the potential to have a significant impact on public health.

## Appendix

Multimedia Appendix 1 Food Hero screen shot: Consent and personal information form.

Multimedia Appendix 2 Food Hero screen shot: Character selection screen.

Multimedia Appendix 3 Food Hero screen shot: Home screen of the private version.

Multimedia Appendix 4 Food Hero screen shot: Home screen of the social version.

Multimedia Appendix 5 Food Hero screen shot: Popup message (exists only for the social version).

Multimedia Appendix 6 Food Hero screen shot: Feeding screen (guided by nutritional information cards and status bars).

Multimedia Appendix 7 Food Hero screen shots: Example of menu assembly process for a full day (including three meals and one snack).

Multimedia Appendix 8 Food Hero screen shot: Menu assembly feedback screen.

Multimedia Appendix 9 Food Hero screen shots: Different examples of menu assembly feedback screens (according to different outcomes).

Multimedia Appendix 10 Food Hero screen shot: Accessories shop screen.

Multimedia Appendix 11 Food Hero screen shot: Sport challenges selection screen.

Multimedia Appendix 12 Food Hero screen shots: Different sport challenges (running, swimming and cycling challenges).

Multimedia Appendix 13 Food Hero screen shot: Quiz screen.

Multimedia Appendix 14 Final Questionnaire.

## Abbreviations

BMI: Body Mass Index
M-W: Mann-Whitney test
SNS: social networking site
