# Supplementary figures and images for: Effects of Social Network Exposure on Nutritional Learning: Development of an Online Educational Platform
Source: JMIR Serious Games. 2015 Oct 5;3(2):e7. doi: 10.2196/games.4002 (PMC4704885; doi:10.2196/games.4002)

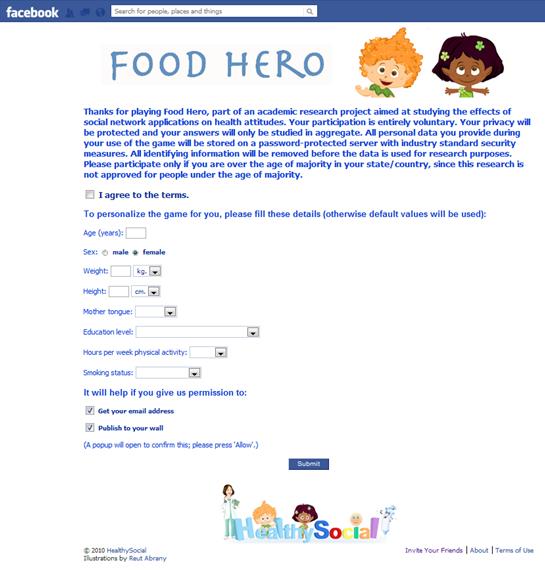

Supplement: Multimedia Appendix 1 [file games_v3i2e7_app1.jpg]

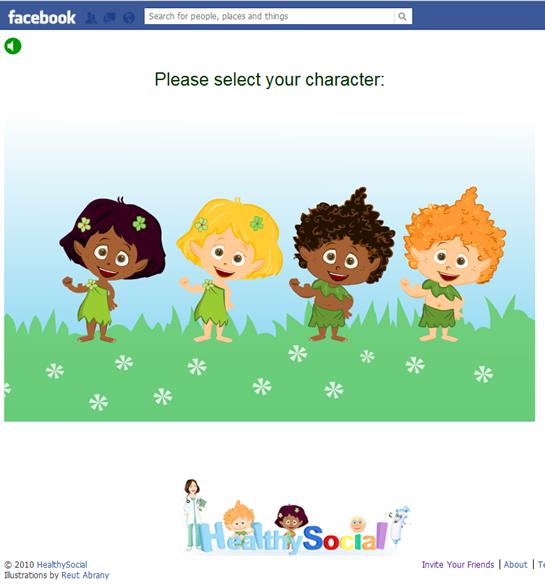

Supplement: Multimedia Appendix 2 [file games_v3i2e7_app2.jpg]

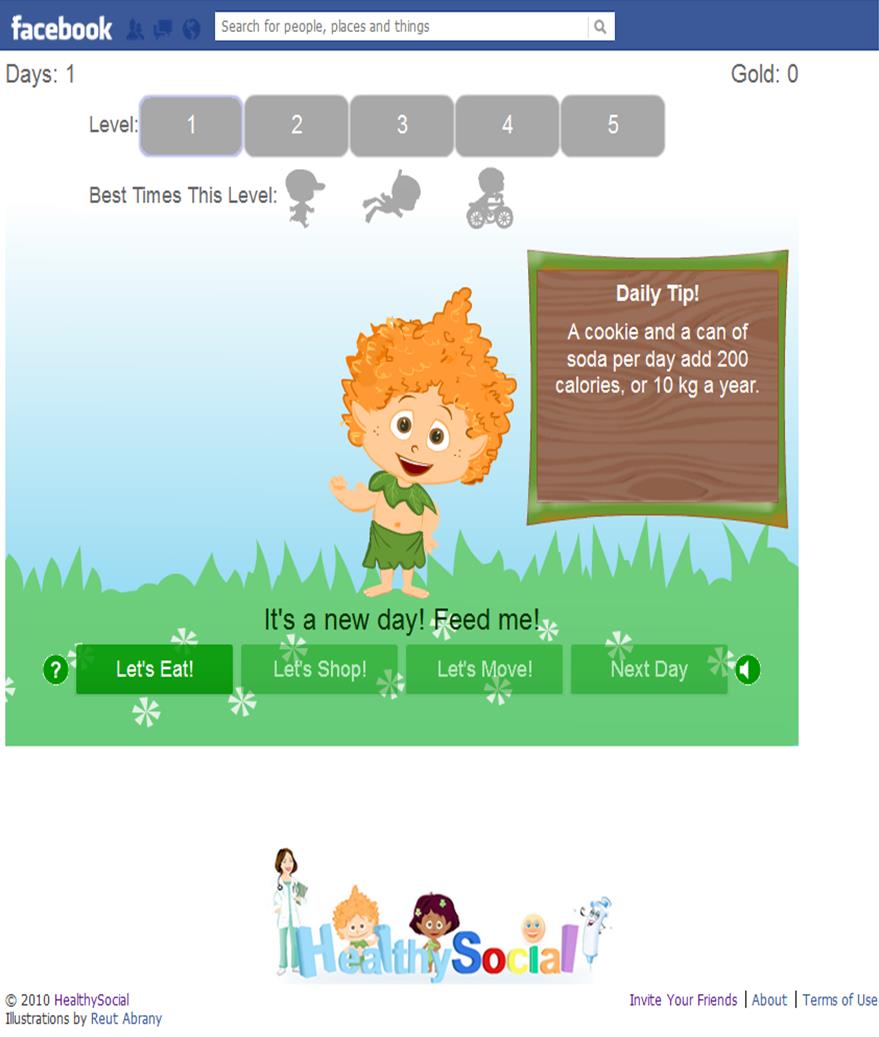

Supplement: Multimedia Appendix 3 [file games_v3i2e7_app3.jpg]

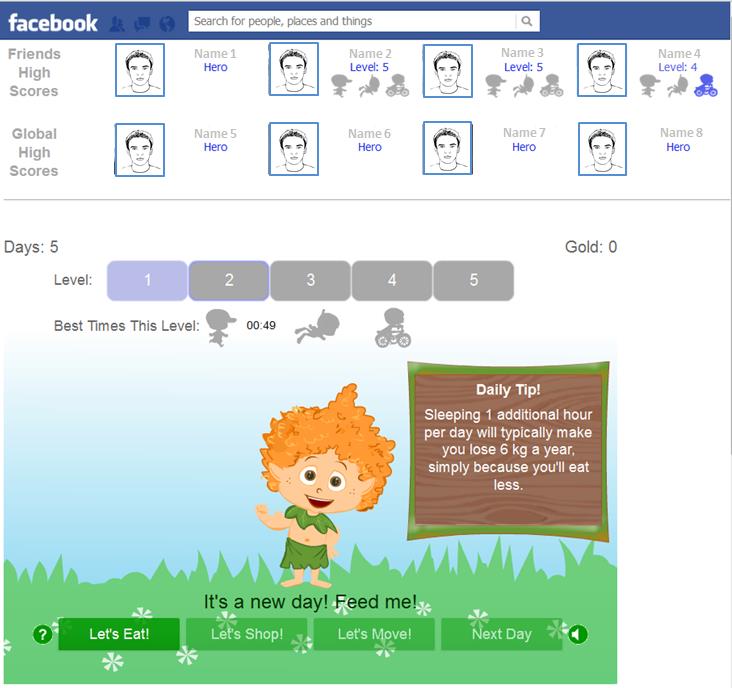

Supplement: Multimedia Appendix 4 [file games_v3i2e7_app4.jpg]

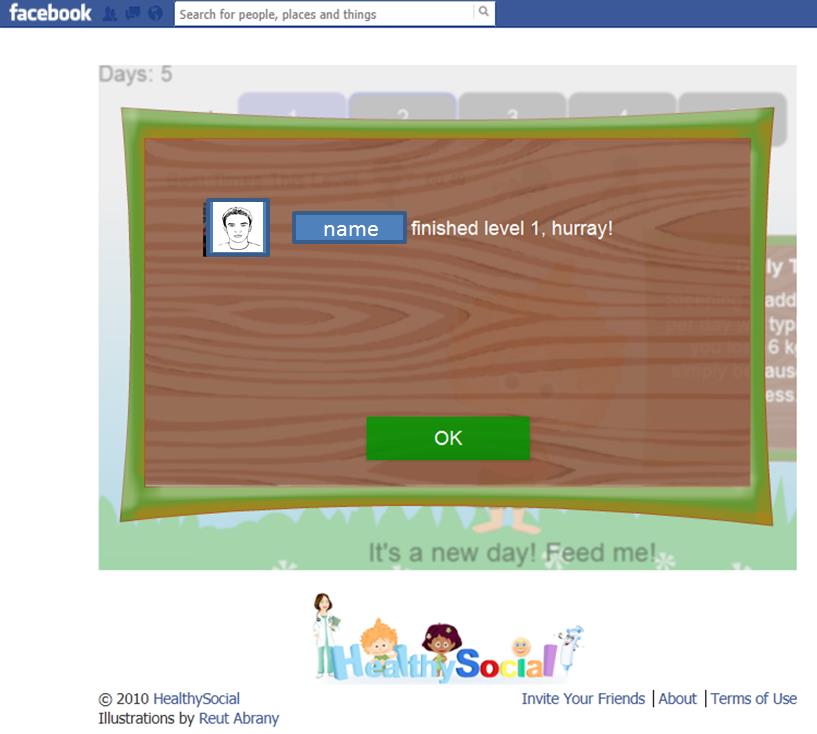

Supplement: Multimedia Appendix 5 [file games_v3i2e7_app5.jpg]

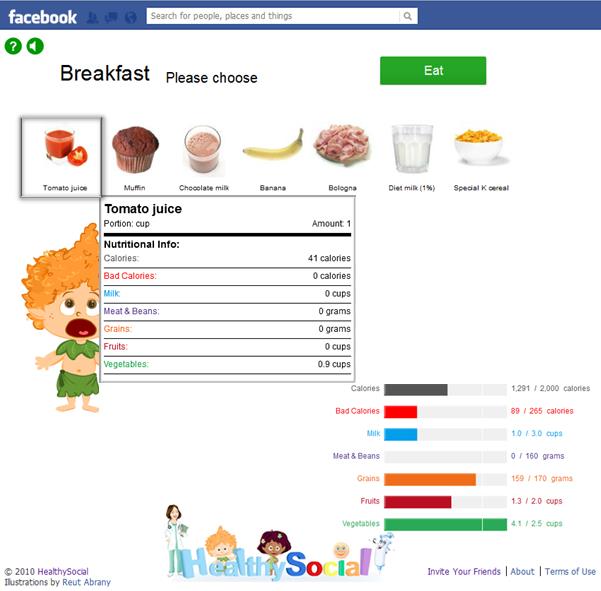

Supplement: Multimedia Appendix 6 [file games_v3i2e7_app6.jpg]

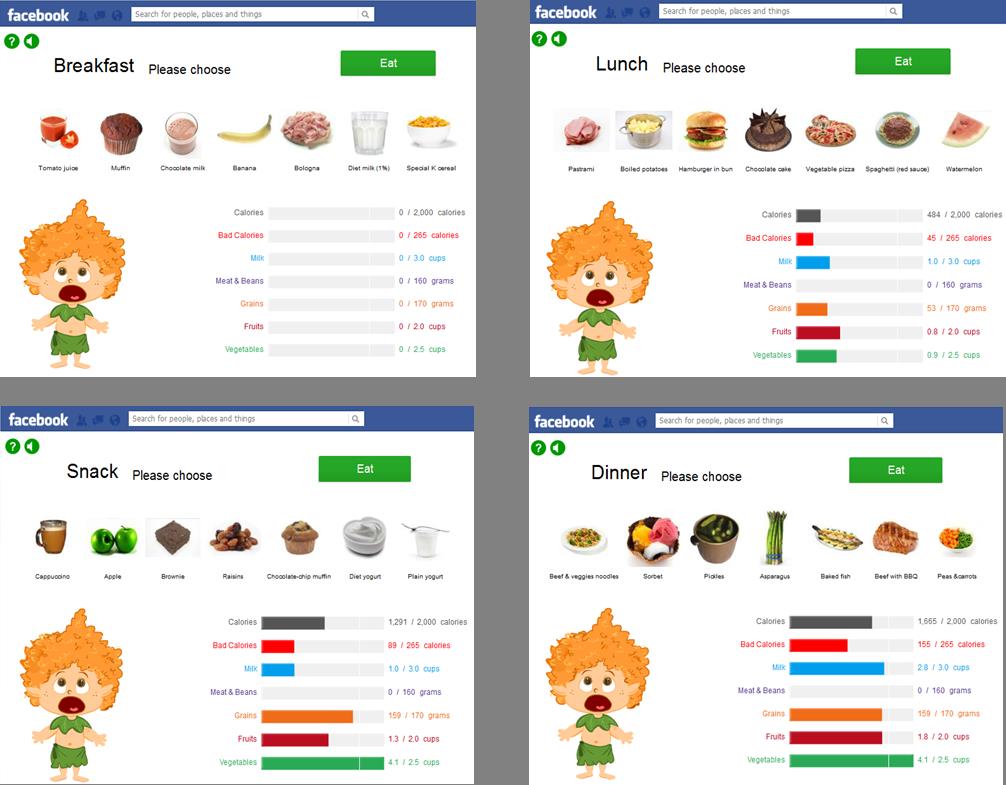

Supplement: Multimedia Appendix 7 [file games_v3i2e7_app7.jpg]

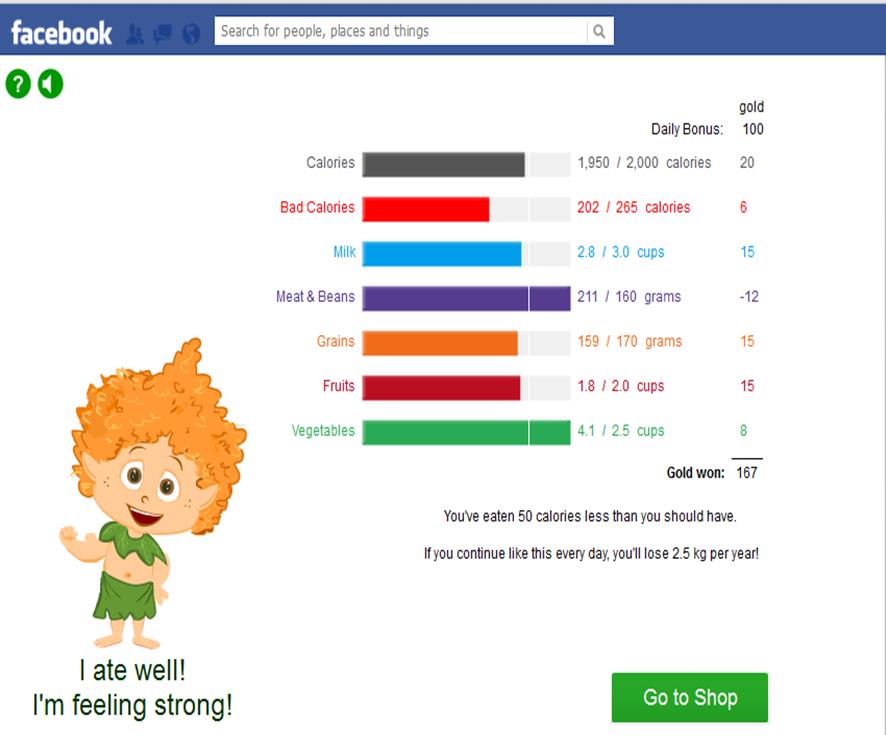

Supplement: Multimedia Appendix 8 [file games_v3i2e7_app8.jpg]

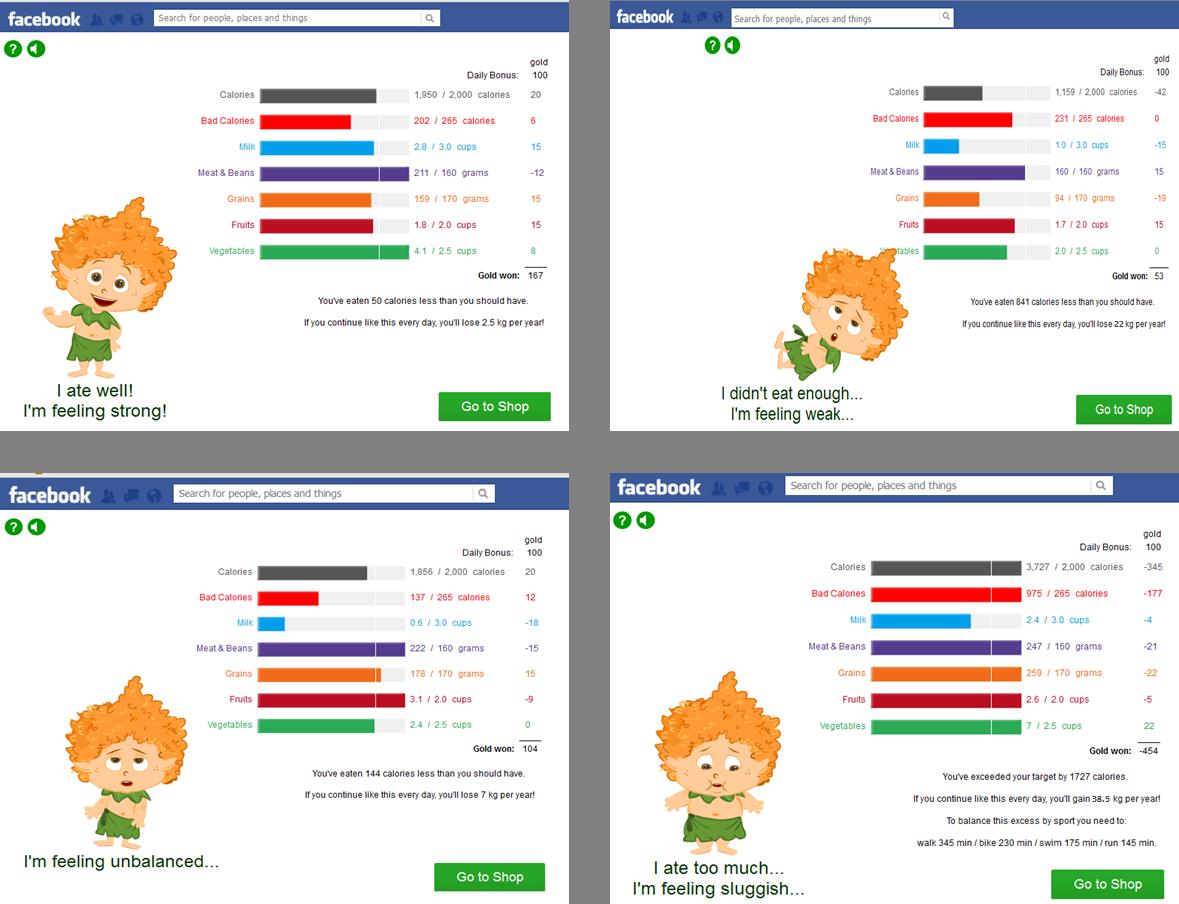

Supplement: Multimedia Appendix 9 [file games_v3i2e7_app9.jpg]

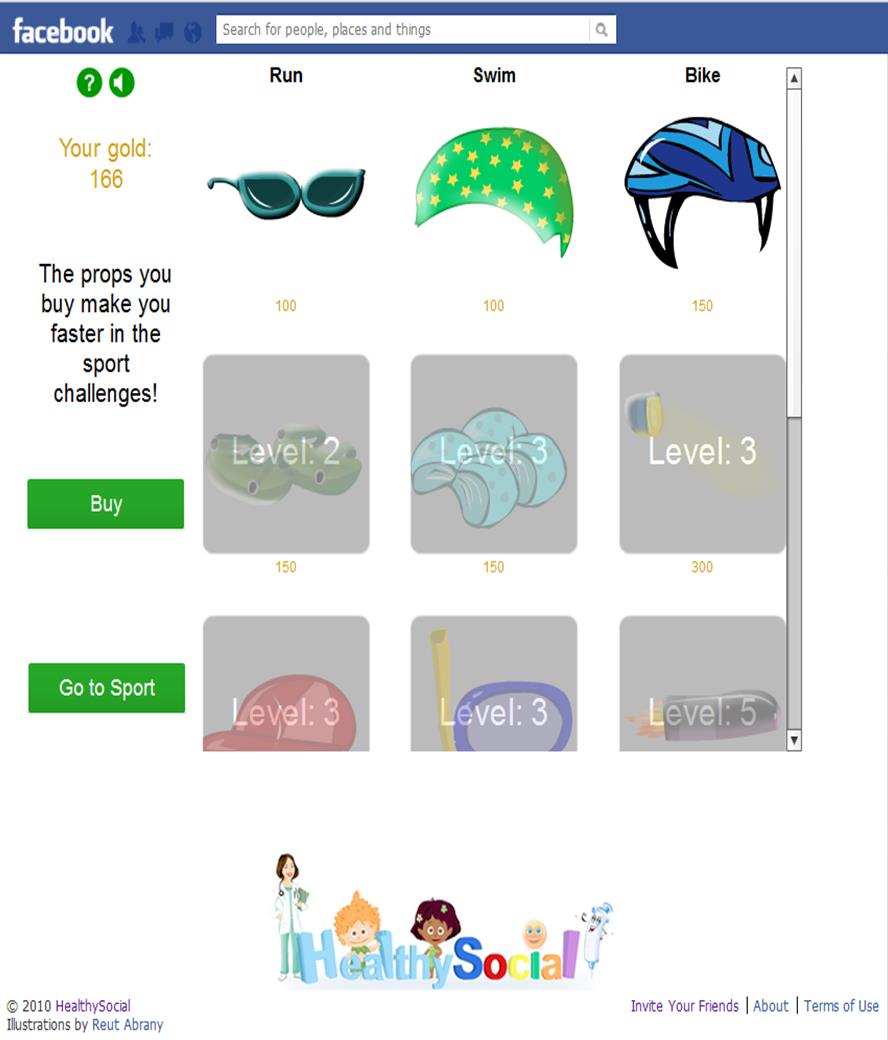

Supplement: Multimedia Appendix 10 [file games_v3i2e7_app10.jpg]

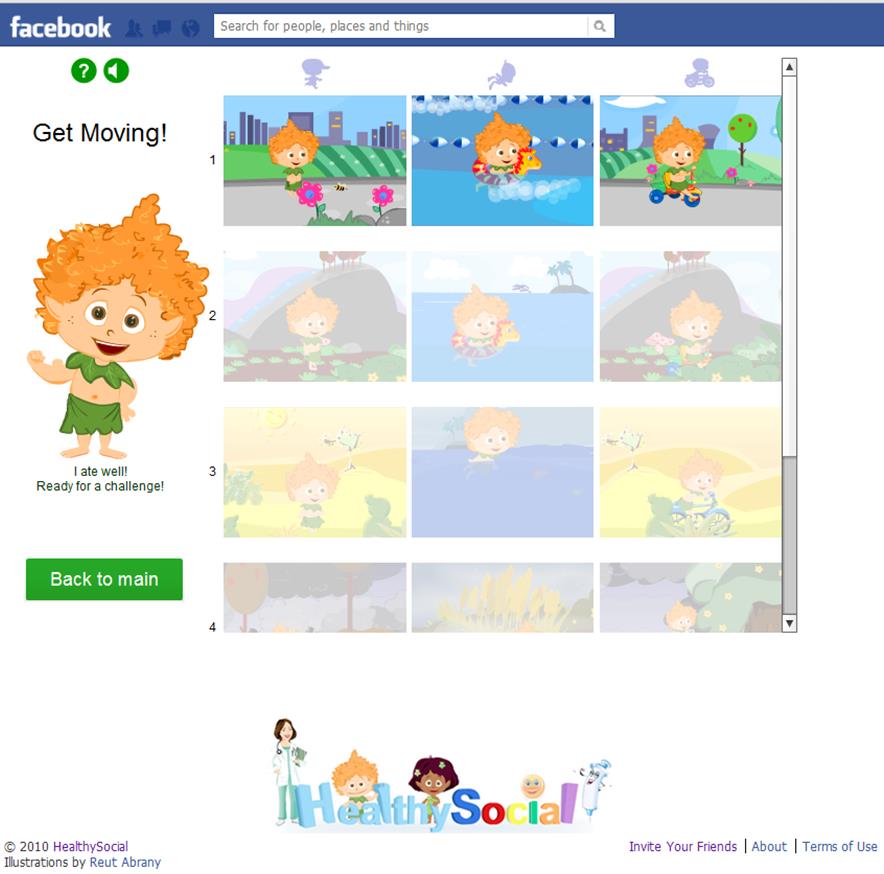

Supplement: Multimedia Appendix 11 [file games_v3i2e7_app11.jpg]

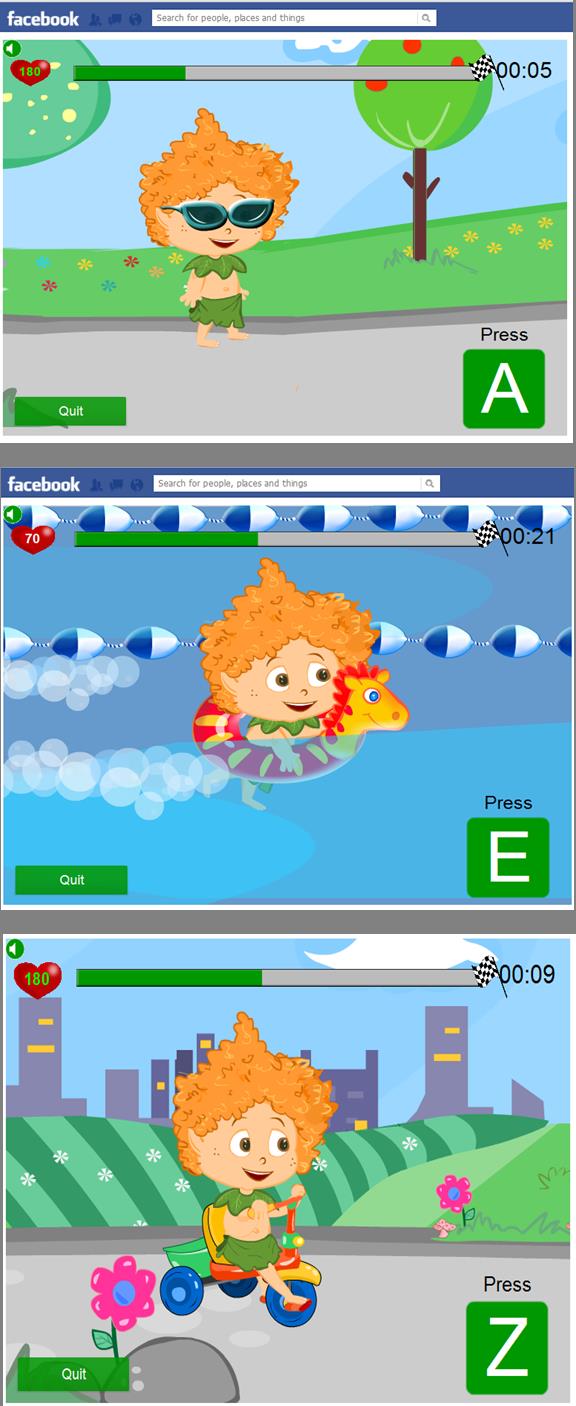

Supplement: Multimedia Appendix 12 [file games_v3i2e7_app12.jpg]

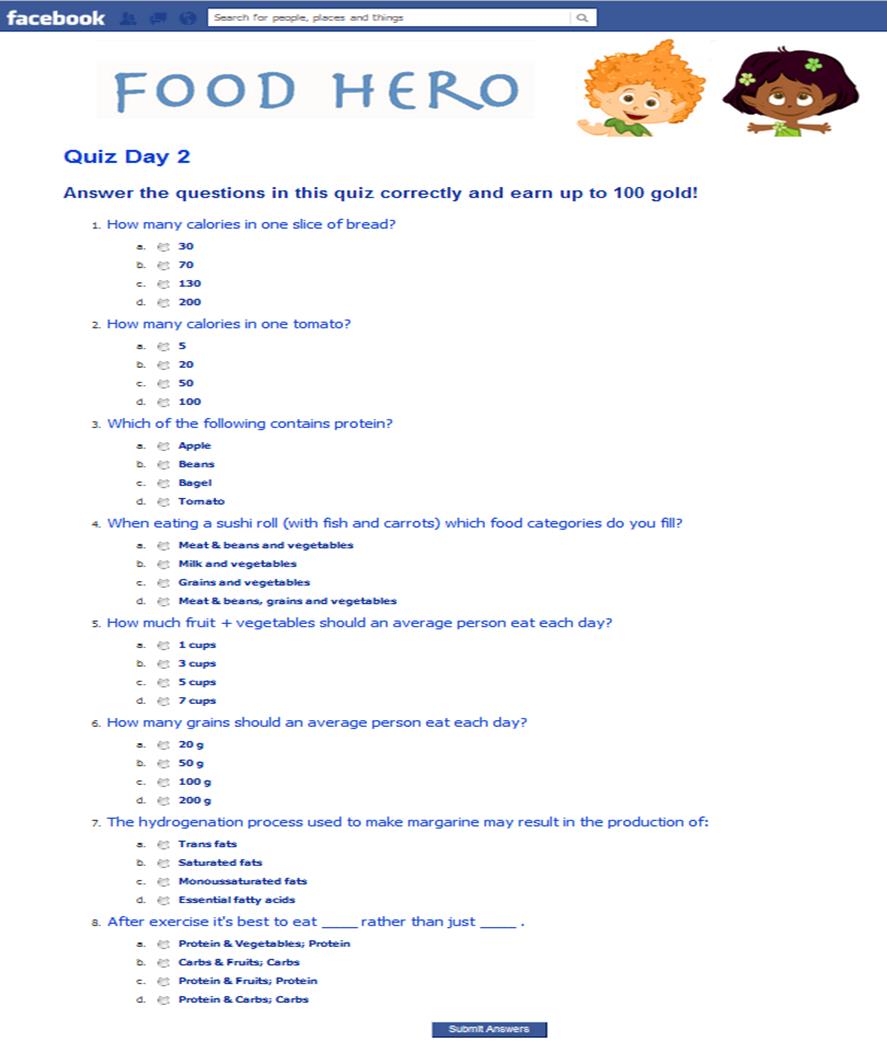

Supplement: Multimedia Appendix 13 [file games_v3i2e7_app13.jpg]
