# Supplementary material for: Effects of Social Network Exposure on Nutritional Learning: Development of an Online Educational Platform
Source: JMIR Serious Games. 2015 Oct 5;3(2):e7. doi: 10.2196/games.4002 (PMC4704885; doi:10.2196/games.4002)
Supplement: Multimedia Appendix 14 [file games_v3i2e7_app14.pdf]

Final Questionnaire

The following is the text of the final questionnaire presented to aprticpants at the end of the followup period:

Thank you for completing the following brief and anonymous questionnaire.

Did playing *Food Hero* positively affect you in any way? Indicate each aspect below:

(No effect; slight effect; medium effect; strong effect; very strong effect)

- It affected my desire to change my eating habits.
- It affected the attention I pay to what I eat.
- It affected the attention I pay to how many calories are in the food I eat.
- It affected my eating habits.
- It affected my physical activity.

If playing the game affected your eating or physical activity habits, please describe in what way it did so:

____
____
____

To what extent do you agree with the following statements?

(strongly disagree; disagree; neither agree nor disagree; agree; strongly agree)

For users with "social" version:

- I followed my friends' performance on Food Hero.
- Seeing my friends' performance in the game increased my motivation to invest more in playing the game in order to succeed.
- Seeing my friends' performance in the game increased my motivation to play the game.
-

For users with "private" version:

- If I had the ability to see my friends' performance in the game, I would look at it.
- If I had the ability to see my friends' performance in the game, I would be more motivated to invest more in playing the game in order to succeed.
- If I had the ability to see my friends' performance in the game, I would be less motivated to invest more in playing the game in order to succeed.
-

How would you improve the game?

____

In what ways could the game be changed in order to make you improve your eating and physical activity habits?

____

Other comments:

____
